# Nuts and their Effect on Gut Microbiota, Gut Function and Symptoms in Adults: A Systematic Review and Meta-Analysis of Randomised Controlled Trials

**DOI:** 10.3390/nu12082347

**Published:** 2020-08-06

**Authors:** Alice C. Creedon, Estella S. Hung, Sarah E. Berry, Kevin Whelan

**Affiliations:** Department of Nutritional Sciences, King’s College London, London SE1 9NH, UK; alice.creedon@kcl.ac.uk (A.C.C.); Estella.Hung@phe.gov.uk (E.S.H.); sarah.e.berry@kcl.ac.uk (S.E.B.)

**Keywords:** nuts, almond, walnut, pistachio, microbiome, microbiota, diversity, gut function, gut symptoms, adults

## Abstract

Nuts contain fibre, unsaturated fatty acids and polyphenols that may impact the composition of the gut microbiota and overall gut health. This study aimed to assess the impact of nuts on gut microbiota, gut function and gut symptoms via a systematic review and meta-analysis of randomised controlled trials (RCTs) in healthy adults. Eligible RCTs were identified by systematic searches of five electronic databases, hand searching of conference abstracts, clinical trials databases, back-searching reference lists and contact with key stakeholders. Eligible studies were RCTs administering tree nuts or peanuts in comparison to control, measuring any outcome related to faecal microbiota, function or symptoms. Two reviewers independently screened papers, performed data extraction and risk of bias assessment. Outcome data were synthesised as weighted mean difference (WMD) or standardised mean difference (SMD) using a random effects model. This review was registered on PROSPERO (CRD42019138169). Eight studies reporting nine RCTs were included, investigating almonds (*n* = 5), walnuts (*n* = 3) and pistachios (*n* = 1). Nut consumption significantly increased *Clostridium* (SMD: 0.40; 95% CI, 0.10, 0.71; *p* = 0.01), *Dialister* (SMD: 0.44; 95% CI, 0.13, 0.75; *p* = 0.005), *Lachnospira* (SMD: 0.33; 95% CI, 0.02, 0.64; *p* = 0.03) and *Roseburia* (SMD: 0.36; 95% CI, 0.10, 0.62; *p* = 0.006), and significantly decreased *Parabacteroides* (SMD: −0.31; 95% CI, −0.62, −0.00; *p* = 0.05). There was no effect of nuts on bacterial phyla, diversity or stool output. Further parallel design RCTs, powered to detect changes in faecal microbiota and incorporating functional and clinical outcomes, are needed.

## 1. Introduction

Nuts have well-documented benefits for human health, with recent systematic reviews highlighting their benefits for cardiovascular health [1,2] and glycaemic control [3]. Nuts represent a valuable dietary intervention for targeting cardiometabolic health in the general population. In comparison, less is known about the impact of nuts on gastrointestinal health and the gut microbiota.

Gastrointestinal health is strongly influenced by the composition of the gut microbiota. From birth and throughout the life cycle, the gut microbiota perform roles vital to host health, ranging from education of the immune system, energy harvest from foods that are otherwise indigestible to humans and the production of short-chain fatty acids (SCFAs), the main energy source of intestinal epithelial cells [4]. Conversely, alterations in the composition of the gut microbiota are a feature of functional bowel disorders such as irritable bowel syndrome (IBS) [5] and constipation [6]. The microbiota have also been associated with common gastrointestinal symptoms in healthy people, such as abdominal pain and bloating [7]. Dietary interventions that target the gut microbiota have valuable implications for the maintenance of gastrointestinal health in the general population. 

Nuts have been suggested to have a prebiotic effect on the gut microbiota [8]. A prebiotic is a substrate selectively used by the host microorganisms conferring a health benefit [9]. Early in vitro studies demonstrated the prebiotic effect of almonds [10] and chestnut extract [11] on *bifidobacteria* and *lactobacilli* respectively. The potential mechanisms behind the observed prebiotic effect of nuts relate to their nutrient composition and physical structure. Nuts are rich in fibre and polyphenols, both of which are utilised as substrates by the gut microbiota. The fermentation of fibre by the gut microbiota produces SCFAs such as butyrate, which promote contractility and mucus secretion in intestinal epithelial cells, in part explaining the beneficial effect of the microbiota on gut function [12]. Polyphenols have a bidirectional relationship with the host microbiota, in which the bacteria process polyphenols into absorbable products, and these products modulate the composition of the microbiota [13]. Nuts are also rich in lipids, which have low bioaccesibility as a consequence of intact cell walls that are resistant to digestion providing a physical barrier to lipid digestion in the upper gastrointestinal tract, and as a result the lipid might therefore reach the colon, where they are potentially utilised by the microbiota [14]. The food matrix of nuts might therefore represent a unique method of delivering a rich supply of fermentable nutrients such as fibre, polyphenols and lipids to the gut microbiota. 

Based on the early in vitro findings, several human trials have been conducted to investigate the impact of nut consumption on gastrointestinal health and the gut microbiota, most usually the faecal microbiota. Most interestingly, Liu and colleagues reported an increase in faecal *bifidobacteria* following 6 weeks of consumption of either whole almonds or almond skins in comparison to a commercial prebiotic in their non-randomised clinical trial [15]. The results of subsequent randomised controlled trials (RCTs) have been conflicting, with some reporting an increase [16], decrease [17] or no effect [18,19,20,21] of nut consumption on faecal *bifidobacteria*. In addition, few studies have investigated the impact of nut consumption on gut function or symptoms [18,20,22]. Therefore, the aim of this study was to investigate the impact of nut consumption on the gut microbiota, gut function, and gut symptoms in healthy adults via a systematic review and meta-analysis of RCTs. 

## 2. Materials and Methods 

This systematic review and meta-analysis was conducted in accordance with the recommendations of the Cochrane Handbook for Systematic Reviews of Interventions [23] and reported in accordance with the Preferred Reporting Items for Systematic Reviews and Meta-analyses (PRISMA) [24]. The eligibility criteria, search strategy, and methodology for screening, data extraction and data synthesis were specified in advance by study authors ACC, SEB and KW and documented in a protocol (published on PROSPERO; CRD42019138169) prior to conducting literature searches. 

### 2.1. Eligibilty Criteria

Eligible studies were RCTs investigating the impact of nut consumption in comparison to control on outcomes related to gut health. The full eligibility criteria for studies included in the review are outlined in Table 1. There were no restrictions for language, publication or date of included studies. 

### 2.2. Search Strategy

Studies were identified by a systematic search of electronic databases, scanning of references lists of eligible papers, hand searching of conference abstracts from the past 5 years and clinical trials databases and consultation with stakeholders.

The following 5 electronic databases were searched: MEDLINE (from 1946 to August 2019; Ovid platform); EMBASE (1974 to August 2019; Ovid platform); Web of science (from 1900 to August 2019; Web of Knowledge platform); CENTRAL (all years; The Cochrane library) and CINAHL (from 1946 to August 2019; EBSCO platform). The search strategy was developed by ACC, SEB and KW and searches were conducted by ACC. Combinations of the following search terms were used to search all databases both as medical subject heading and free text terns: nut*; almond; brazil nut; cashew; chestnut; hazelnut; macadamia; marking nut; pecan; pine nut; pistachio; walnut; groundnut; peanut; gut microbiome; short-chain fatty acids; stool pH; gut transit; stool frequency; stool consistency; gut symptoms. The search also included the Latin names of all nuts listed. A detailed search strategy is included in Supplementary Note S1: Example search strategy for MEDLINE (Ovid). The final search was run on 29 July 2019. 

Abstracts from the following conferences were hand searched: The Nutrition Society (2015–2019; *Proceedings of the Nutrition Society*), The American Society for Nutrition (2018–2019; *Current Developments in Nutrition*), and The European Nutrition conference (2017; *Annals of Nutrition and Metabolism*). Two clinical trials databases, The World Health Organisation International Standardised Randomised Control Trial Number registry (www.isrctn.com) and the US National Institutes of Health (www.clinicaltrials.gov), were searched to identify any completed, but unpublished studies. Experts in the field and industry bodies were contacted and the reference lists of all eligible papers were back-searched to identify unpublished data or studies that were absent from the electronic search. 

### 2.3. Screening

References were exported to a reference manager (EndNoteX7; Thomson Reuters) and duplicates were removed automatically by the software and by hand. Two reviewers (ACC and ESH) independently screened titles and abstracts in a blinded standardised manner. Full text articles of potentially eligible studies were obtained online. Where full text articles were not available online authors were contacted by email. Two reviewers (ACC and ESH) screened all available full text articles against the inclusion and exclusion criteria. Disagreements between reviewers were resolved at all stages by consultation with a third reviewer (KW). The per cent agreement between reviewers at each stage was recorded. 

### 2.4. Data Extraction 

A standard data extraction form was developed to record data from eligible studies on participant characteristics, interventions, comparators, outcomes and study design (Table 1). Two reviewers (ACC and ESH) independently extracted data from included studies. Where there was insufficient information available to complete data extraction, the corresponding author of the study was contacted. Disagreements were resolved by discussion between review authors. Where no consensus could be reached, a third reviewer (KW) was consulted. 

Two reviewers (ACC and ESH) independently assessed the validity of included studies using the Cochrane Collaboration tool for assessing risk of bias [26]. The tool assesses validity of studies based on six domains (selection bias, performance bias, detection bias, attrition bias, reporting bias and other bias). Judgments on risk of bias related to each domain were categorized as “low”, “high” or “unclear” in line with the Cochrane guidance. Where there was insufficient information to complete the assessment, this information was sought from the corresponding study author by email.

### 2.5. Data Synthesis

Meta-analysis was performed where ≥2 studies reported the same outcome. Data were entered into RevMan version 5.3 software (The Cochrane Collaboration), which was used to conduct meta-analyses. As the majority of outcome variables of interest consisted of continuous data, difference in means was the primary measure of intervention effect. Where an outcome was measured and reported in the same way across studies, weighted mean difference (WMD) was used. When studies reported outcomes that were not directly comparable due to differences in measurement or reporting, standardised mean difference (SMD) was used. All meta-analyses were performed using a random effects model. For crossover studies, data for the intervention and control periods was entered separately [23]. For trials with multiple intervention arms, each arm was treated as a separate study in the meta-analysis, whereby each arm was compared to the control group independently. The sample size of the control group was divided by the number of intervention arms to reduce the effect size error, as recommended [23]. Forest plots were generated for all outcomes. 

To determine heterogeneity the chi-square test was used and quantified by the *I*^2^ statistic. Values of 50% and 75% were used to define substantial and considerable heterogeneity respectively [23]. Where results are significant or heterogeneity was high, sensitivity analyses were conducted. The following predefined sub-group analyses were planned to investigate differences by: (1) participant group (healthy vs. clinical participants); (2) type of nut (almond, walnut, pistachio); (3) duration of intervention (greater or less than 4 weeks); (4) dose of intervention (greater or less than 45 g per day); (5) method of measurement of microbiota (culture vs. DNA based techniques); and (6) study design (crossover vs. parallel). 

## 3. Results

**Figure 1:**
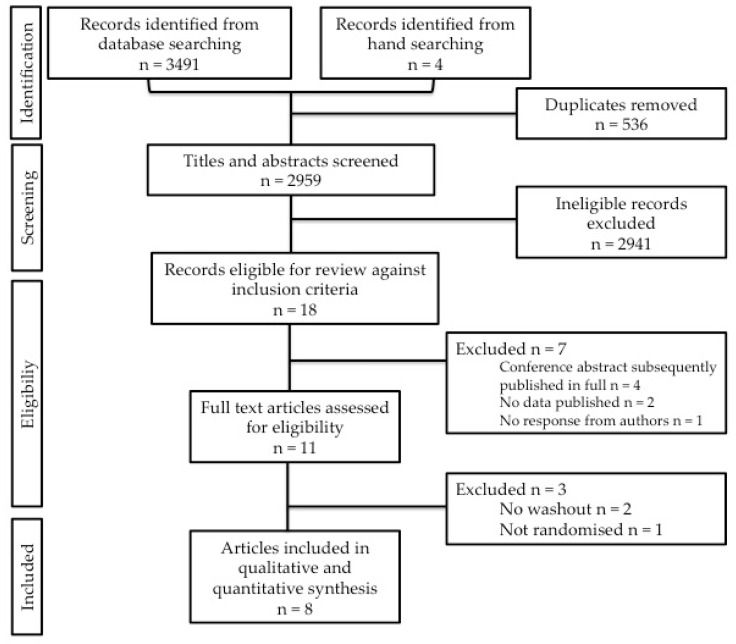
PRISMA flow diagram of studies in systematic review. PRISMA, Preferred Reporting Items for Systematic Reviews and Meta-Analyses.

A total of 2955 non-duplicated records were identified following the electronic search and an additional four records were identified in manual searches. Following scanning of titles and abstracts 18 of the records were deemed potentially eligible for inclusion, four of which were excluded as they were conference abstracts that had been subsequently published in full and already included in the identified records. The authors of three clinical trials registrations identified in the search were contacted to provide data for screening, in two cases the data was yet to undergo analysis, and the authors of the third record did not respond so these records were also excluded. For the remaining 11 records, full text articles were retrieved and assessed by reviewers independently. In total eight articles, reporting nine RCTs fulfilled the criteria for inclusion in the review. Reviewers agreed on inclusion of 7/8 articles, representing 89% agreement. Numbers identified and reasons for exclusion of articles at each stage are presented in Figure 1.

### 3.1. Study Characteristics

The characteristics of eligible studies are outlined in Table 2. One article investigated two different nut types in two separate crossover trials (almonds and pistachios) [20]. Each trial consisted of two intervention arms investigating a high dose (84 g/d) or a low dose (42 g/d) of nuts compared with a control diet devoid of nuts. Each arm (high dose, low dose) of each trial (almond, pistachio) was entered into the meta-analyses separately in comparison to the control arm. A second article consisted of four intervention arms (whole natural almonds, whole roasted almonds, chopped almonds and almond butter) and a single control arm [19]. Each intervention arm of the trial was entered into the meta-analysis separately in comparison to the control arm. Finally, one trial did not report any outcome data in a form that could be meta-analysed [22]. The authors were contacted but supplied no further data. As a result, 14 separate interventions were included in the meta-analyses, with a total of 356 participants. 

Eight RCTs measured outcomes related to faecal microbiota including relative abundance at the phyla and genus levels, and metrics for alpha and beta diversity [16,17,18,19,20,21,27]. Three RCTs reported stool frequency [18,20,22] and two RCTs reported gut symptoms [18,22]. No RCTs measured gut transit time or stool form. In line with the recommendations in The Cochrane Handbook, due to the small number of studies included in the meta-analyses, and the fact that data from several studies was crossover and therefore not independent, subgroup analyses were not conducted [23]. To investigate heterogeneity, or to test for robustness where the meta-analyses were significant, sensitivity analyses were conducted and are discussed where ≥2 studies or intervention arms contributed to the analysis.

### 3.2. Outcomes 

#### 3.2.1. Summary of Meta-Analyses 

The outcome of each meta-analysis is summarised in Table 3.

#### 3.2.2. Methods of Analysis and Reporting of Gut Microbiota

There were differences in analysis and reporting methods between the eight RCTs included in the meta-analyses. Methods of DNA extraction varied, with three studies using the MoBio power soil DNA isolation kit [17,19,21], one study using the QIAmp East DNA Stool Kit [18], one study using the Qiagen Powersoil DNA isolation kit [27], and three studies following previously published protocols with modifications [16,20]. Primers used to target the 16S gene also varied with three studies targeting the V4 region [17,19,27], three studies targeting V1–V3 regions [18,20], one study targeting the V3/4 regions [16], and one targeting the V4/5 regions [21]. The reads following sequencing were reported as ‘reads per sample’ in one study (88,880 [17]), ‘reads per sample following quality filtering’ in two studies (28,680 and 33,194 respectively; [18,19]), and ‘total reads following quality filtering’ in four studies (range 3259–3008,191 [20,21,27]). One study did not report reads obtained [16]. When assigning taxonomy, five studies used the 13_8 Greengenes database [17,19,20,21], one study used the Silva 132 database [27], while two studies did not report this information [16,18]. Finally, six studies reported relative abundance of bacterial taxa in terms of operational taxonomic units (OTUs) [16,17,18,19,20], while two studies reported amplicon sequence variants (ASVs) [21,27]. As a result of these methodological variations in analysis and reporting, all microbial outcomes were meta-analysed using SMD as the effect estimate.

#### 3.2.3. Relative Abundance of Bacterial Phyla 

Faecal microbiota at the phylum level were measured in eight RCTs. Corresponding authors of six of the studies were contacted to obtain data for inclusion in the meta-analysis. Of these, five replied, two of who provided data for inclusion in the analyses [18,21], while another provided data that was in a format that could not be entered into the meta-analysis [20]. Data from remaining studies was not obtained despite request [16,27], resulting in a total of four trials, including seven separate interventions for inclusion in the meta-analyses. 

**Figure 2:**
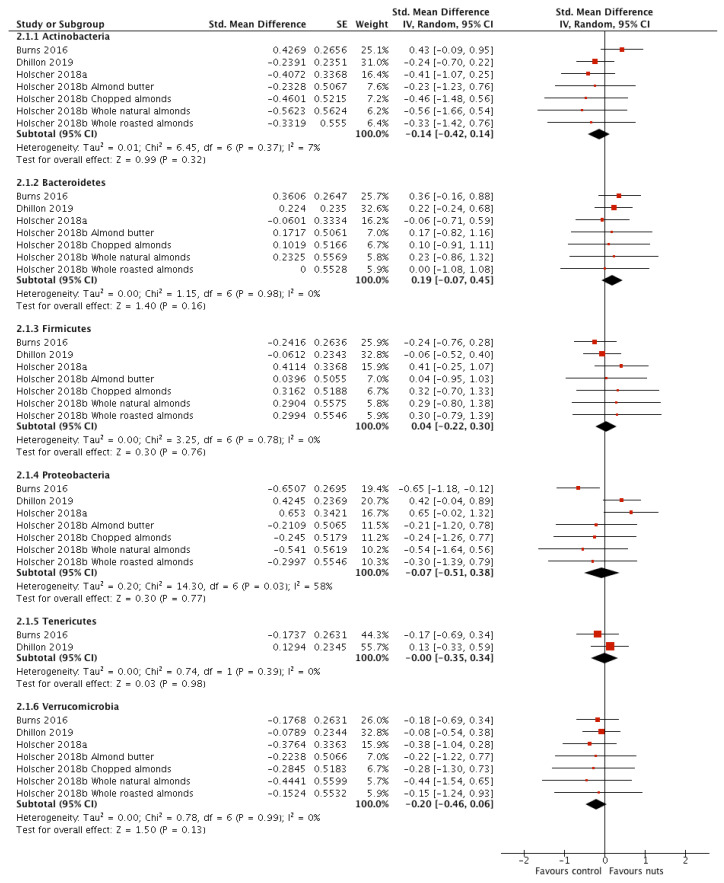
Forest plot of relative abundance of bacterial phyla measured in faecal samples of participants taking part in randomised controlled trials comparing nut consumption to control in adults. Meta-analyses were conducted using a random effects model. Values are standardised mean difference (95% confidence interval).

All seven interventions measured and reported the impact of nut consumption on the following phyla: *Actinobacteria*, *Bacteroidetes, Firmicutes, Proteobacteria*, and *Verrucomicrobia*. Overall, the meta-analyses revealed no effect of nut consumption on bacterial phyla (Figure 2), with no significant heterogeneity for any phylum except *Proteobacteria* (*I*^2^ = 58%, *p* = 0.03). As all studies measured abundance of *Proteobacteria* in healthy participants using DNA based techniques, sensitivity analyses were conducted only for nut type, duration and dose of intervention and study design (Table S1: Sensitivity analyses). One study investigated walnuts [17], while the rest investigated almonds [18,19,21]. Removal of the walnut study did not affect significance (SMD: −0.20; 95% CI: −0.66, 0.25; *p* = 0.38) and heterogeneity remained substantial (*I*^2^ = 50%, *p* = 0.08). One of the 4 studies had a duration of intervention >4 weeks, a dose of >45 g/d, and a parallel design [21]. When this study was removed there was no effect on significance (SMD: −0.20; 95% CI, −0.67, 0.28; *p* = 0.42), however the level of heterogeneity was marginally reduced to moderate (*I*^2^ = 47%, *p* = 0.09).

Relative abundance of the phyla *Tenericutes* was investigated in two interventions and when meta-analysed there was no effect of nut consumption [18,21] (Figure 2). 

Of the studies that measured faecal bacterial phyla but could not be included in the meta-analyses, one study presented graphs illustrating that there was no change in predominant phyla following consumption of either almonds or pistachios in comparison to control [20]. Another reported no significant differences in *Bacteroidetes, Firmicutes* or *Proteobacteria* following consumption of walnuts [27], and the final study reported shifts in the relative abundance of predominant phyla from *Firmicutes* (61.2% after walnut diet, 63.9% following control) to *Bacteroidetes* (30.8% following walnut diet, 27.4% following control) that were not statistically significant [16]. 

#### 3.2.4. Relative Abundance of Bacterial Genera 

Eight RCTs measured faecal microbiota at the genus level. The authors of six of the RCTs were contacted for additional data, and all replied. Of these, two studies reported limited data in their publications, but no further data was obtained and so these studies are present in a limited number of meta-analyses of bacterial genera [16,27]. The authors of two studies sent the requested data [21], however for one of the studies, that reported two RCTs, data was provided in a format that could not be entered into the meta-analyses [20]. For the final RCT the data was not obtained [18], resulting in a total of five trials, reporting eight interventions for inclusion in the meta-analyses. 

**Figure 3:**
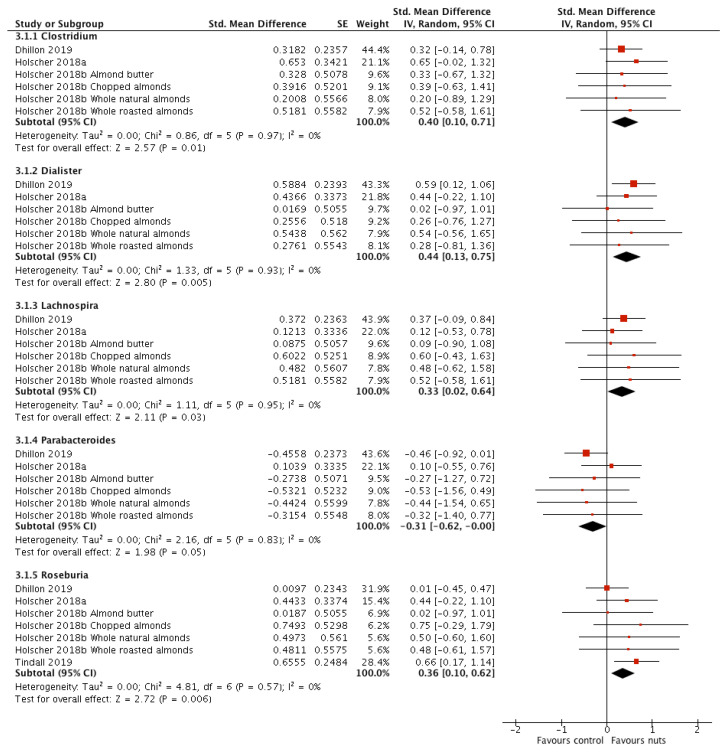
Forest plot of relative abundance of bacterial genera measured in faecal samples of participants taking part in randomised controlled trials comparing nut consumption to control in adults. Meta-analyses were conducted using a random effects model. Only statistically significant differences are shown. Values are standardised mean difference (95% confidence interval).

Nineteen faecal bacterial genera were measured and reported by ≥2 interventions (Table 3). Three studies contributed to the majority of these [17,19,21], one of which contributed four separate interventions resulting in six interventions included in the meta-analyses. Nut consumption significantly increased *Clostridium* (SMD: 0.40; 95% CI, 0.10, 0.71; *P* = 0.01), *Dialister* (SMD: 0.44; 95% CI, 0.13, 0.75; *P* = 0.005), and *Lachnospira* (SMD: 0.33; 95% CI, 0.02, 0.64; *p* = 0.03), and significantly decreased *Parabacteroides* (SMD: −0.31; 95% CI, −0.62, −0.00; *p* = 0.05) (Figure 3). There was no significant heterogeneity observed in these findings. Sensitivity analyses were conducted (Table S1: Sensitivity analyses). All studies investigated healthy adults and measured bacterial relative abundance using DNA based techniques. One intervention investigated walnuts [17]. When this study was removed, statistical significance was lost for *Clostridium* (SMD: 0.34; 95% CI, −0.01, 0.68; *p* = 0.06) with no effect on heterogeneity. Another study administered nuts for >4 weeks, in a dose >45 g/d, and in a parallel design trial. When this study was removed significance was lost for *Dialister* (SMD: 0.33; 95% CI, −0.08, 0.74; *p* = 0.12), *Lachnospira* (SMD: 0.30; 95% CI, −0.11, 0.71; *p* = 0.15), and *Parabacteroides* (SMD: −0.20; 95% CI, −0.61, 0.21; *p* = 0.35) with low heterogeneity for all results. 

One additional study contributed to the meta-analyses of Faecalibacterium, Roseburia, and Streptococcus [27], resulting in seven interventions in the meta-analyses. Of these, only Roseburia was significantly affected by nut consumption, (SMD: 0.36; 95% CI, 0.10, 0.62; *p* = 0.006) with no evidence of heterogeneity (Figure 3). Sensitivity analyses for Roseburia (Table S1: Sensitivity analyses) revealed potential effects of participant group (healthy adults only [17,19,21]; SMD: 0.24; 95% CI, −0.06, 0.55; *p* = 0.12) and nut type (almond only [19,21]; SMD: 0.19; 95% CI, −0.16, 0.53; *p* = 0.29; walnut only [17,27]; SMD: 0.58; 95% CI, 0.19, 0.97; *p* = 0.004), with no evidence of heterogeneity. Two studies in the meta-analysis administered nuts for <4 weeks at a dose of <45 g/d (SMD: 0.43; 95% CI, 0.02, 0.84; *p* = 0.04) [17,19], with no heterogeneity observed for the result. The remaining two studies administered nuts for >4 weeks at a dose of >45 g/d (SMD: 0.33; 95% CI, −0.31, 0.96; *p* = 0.31) [21,27], however substantial heterogeneity was detected (I^2^ = 72%; *p* = 0.06). 

*Bifidobacteria* was investigated in four studies [16,17,19,21], one of which contributed four intervention arms resulting in a total of seven interventions. There was no effect of nut consumption on *Bifidobacteria* (SMD: −0.09; 95% CI, −0.39, 0.21; *p* = 0.56). Moderate heterogeneity was detected (*I*^2^ = 36%; *p* = 0.16), which appeared to be explained by nut type and duration of intervention, however there was no impact on significance (Table S1: Sensitivity analyses). 

Of the studies that measured faecal bacterial genera but could not be included in the meta-analyses, one reported genus level shifts following nut consumption but no further details were provided [18], while another reported a decrease in numbers of lactic acid bacteria following pistachio consumption, and that *Bifidobacteria* was unaffected by consumption of either almonds or pistachios [20]. 

#### 3.2.5. α-Diversity

Eight RCTs measured α-diversity metrics and the corresponding authors of six trials were contacted. In three cases the requested data was obtained [17,18,19], and in the remaining cases it was not [20,27], resulting in five RCTs, reporting eight separate interventions included in the meta-analyses. 

**Figure 4:**
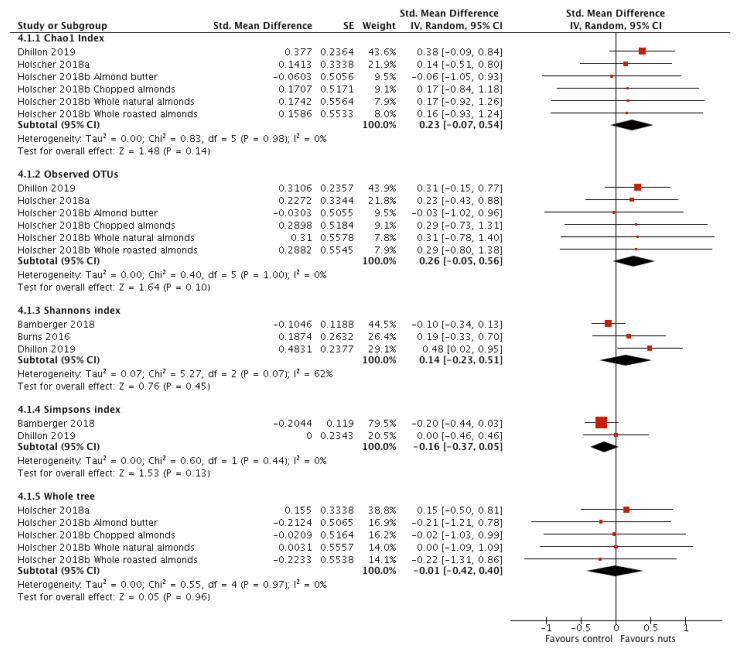
Forest plot of α-diversity metrics measured in faecal samples of participants taking part in randomised controlled trials comparing nut consumption to control in adults. Meta-analyses were conducted using a random effects model. Values are standardised mean difference (95% confidence interval).

The following α-diversity metrics were measured in ≥2 studies and underwent meta-analysis: Chao1 index [17,19,21]; observed OTUs [17,19,21]; Shannon index [16,18,21]; Simpson index [16,21]; Whole tree phylogenetic diversity [17,19] (Figure 4). There was no significant impact of nut consumption on any α-diversity metric. Heterogeneity was substantial in the meta-analysis of Shannon index (*I*^2^ = 62%; *p* = 0.07) and sensitivity analyses were conducted (Table S1: Sensitivity analyses). All studies were conducted in healthy adults, using DNA based techniques. One study investigated walnuts [16], while the other two studies investigated almonds [18,21]. Removal of the walnut study revealed that almond consumption increased Shannon index, though this was borderline statistically significant (SMD: 0.35; 95% CI 0.00, 0.70; *p* = 0.05), and this addressed the heterogeneity (*I*^2^ = 0%; *p* = 0.40). Sensitivity analysis of intervention duration had no impact on the result. Finally, one study administered nuts at a dose >45 g/d and was of parallel design [21], while the remaining two administered <45 g/d and were of crossover design [16,18]. Removal of the former study addressed the heterogeneity but had no impact on the result for Shannon index (SMD: −0.05; 95% CI, −0.27, 0.17; *p* = 0.63; *I*^2^ = 2%; *p* = 0.31).

Amongst studies that measured α-diversity but were not included in the meta-analysis, one reported no difference in observed species following walnut consumption in comparison to control [27]. The second study, which included two separate RCTs, reported no effect of pistachio or almond consumption on Chao1 index [20]. 

#### 3.2.6. β-Diversity

Eight RCTs measured and reported β-diversity. In dietary studies, β-diversity is a measure of overall change in bacterial communities between samples from the same participant [28]. It is commonly reported graphically in order to visualise any clustering of samples that may represent an effect of the intervention. As a result, no studies reported or provided data on β-diversity metrics in a format suitable for meta-analysis.

Of the studies investigating almonds, two reported no significant effect of almond consumption on β-diversity [19,21], and one did not report any findings for this outcome, despite measuring it [18]. Another study that included two separate trials investigating consumption of almonds or pistachios reported that pistachios had a greater effect on β-diversity than almonds, but the data was not reported [20]. 

Of studies investigating walnuts, analysis of UniFrac distances between samples revealed a significant effect of walnut consumption on β-diversity in two studies ([16], *p* = 0.02; [17], *p* = 0.03). In contrast another study reported no distinct clustering of samples by diet, indicating no effect of walnuts in comparison to control [27].

#### 3.2.7. Stool Output

Three studies, including four RCTs, measured stool frequency [18,20,22]. One study reported stool frequency on a three-point scale (mild, moderate, severe) with no details on cut-offs used for each category [22]. Corresponding authors were contacted but data was not obtained and so this study was not included in the meta-analysis. 

**Figure 5:**
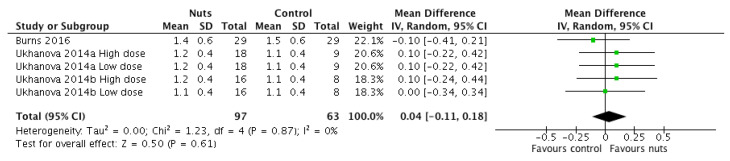
Forest plot of stool frequency (*n*/day) of participants taking part in randomised controlled trials comparing nut consumption to control in adults. Meta-analyses were conducted using a random effects model. Values are weighted mean difference (95% confidence interval).

Of the remaining three RCTs, two provided nuts in two doses (42 g/d, 84 g/d), which were entered into the meta-analysis separately [20]. As a result, five interventions were included in the meta-analysis. One study reported stool frequency in the units mean number per week (n/wk) [18], in the other studies it was reported as mean number per day (n/d) [20]. For the former study n/wk and its standard error were both divided by seven to calculate n/d and its standard error. Stool frequency was then meta-analysed using weighted mean difference as the effect estimate. Overall, nut consumption had no effect on stool frequency per day (WMD: 0.04; 95% CI, −0.11, 0.18; *p* = 0.61) and there was no heterogeneity detected (Figure 5). 

#### 3.2.8. Gastrointestinal Symptoms

Two studies evaluated gastrointestinal symptoms [18,22], however these were not meta-analysed due to differences in measurement method and outcome reporting. The first study measured the impact of almonds on gastrointestinal symptoms in healthy adults using the gastrointestinal symptoms ratings scale (GSRS). The authors grouped symptoms into 5 syndromes (diarrhoea, constipation, abdominal pain, indigestion, reflux) and participants scored symptoms from 1 (no discomfort) to 7 (severe discomfort); with the symptoms scores within a syndrome averaged to give the total score for that syndrome. Almond consumption resulted in a significant decrease in scores for diarrhoea and reflux compared with control [18]. 

The second study measured the effect of almonds on pain intensity, pain frequency and bloating duration in adults with diarrhoea predominant IBS (IBS-D). Participants indicated whether these symptoms were mild/moderate or severe, and the number of participants in each category at baseline and endpoint is reported, however it is unclear over how long the symptoms were measured. Authors reported a significant increase in the numbers of participants in the severe category of all symptoms measured in the almond group in comparison to control [22]. 

### 3.3. Risk of Bias

**Figure 6:**
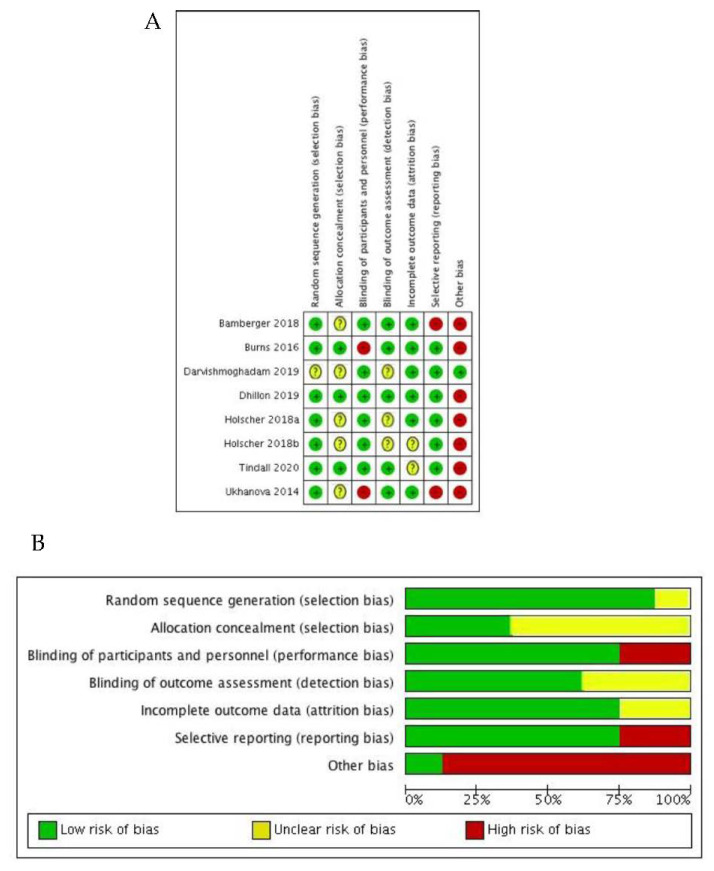
Risk of bias in studies investigating the impact of nut consumption on gut microbiota and gut function. (**A**) Risk of bias in individual studies. (**B**) Risk of bias across studies for each category.

Methodological quality was variable across the included studies (Figure 6). No studies were at a low risk of bias across all categories. In addition, no categories were at a low risk of bias across all studies. 

## 4. Discussion

This systematic review and meta-analysis was conducted to address the hypothesis that nuts have a prebiotic effect on the gut microbiota, and that this may benefit gut function and gut symptoms. The systematic search of the literature identified nine RCTs investigating the impact of nut consumption on the faecal microbiota, stool frequency and/or gut symptoms. Overall, there were no effects of nut consumption on the microbiota at the phylum level, however some genera were significantly altered. Meta-analysis also revealed nut-specific effects on global bacterial diversity measures. Gastrointestinal function has been investigated in few studies, however the current body of evidence suggests no effect of nut consumption on stool output. 

The human gut microbiota is characterised by core communities of bacteria that remain stable over time and have been associated with long-term diet [29]. It is also well established that short-term diet exerts a profound effect on the microbiota composition [30]. The impact of acute changes in dietary patterns, or the introduction of fibre or prebiotics, on gut microbiota composition has been widely reported [31,32,33]. In contrast relatively few studies have been conducted which focus on the impact of whole foods on the gut microbiota composition. One exception is nuts, which have been the subject of several RCTs seeking to modulate gut microbiota composition for the benefit of health. To our knowledge, this is the first systematic review and meta-analysis of the evidence. 

Results of the meta-analysis illustrate a stable faecal microbiota at the phylum level, and sensitivity analyses showed no effect of nut type, dose, duration of intervention or study design. In contrast, several bacteria were modulated by nut consumption at the genus level. The prebiotic effect of nuts was first suggested based on pre-clinical and non-randomised trials that demonstrated a benefit of almond consumption on faecal *Bifidobacteria* [10,15,34,35]. The results of the meta-analysis did not support an effect on *Bifidobacteria*, however high heterogeneity was observed for this result. Sensitivity analyses addressed the heterogeneity but had no effect on the outcome of the meta-analysis. 

Nut consumption increased the relative abundances of the genera *Clostridium, Lachnospira* and *Roseburia*, all of which are known butyrate producers [36,37,38]. Butyrate is vital for gastrointestinal health, both as an energy source for intestinal colonocytes and in the maintenance of the intestinal epithelium [12]. As such, the enrichment of butyrate producers supports the hypothesis of the prebiotic effect of nut consumption. 

Dietary lipids have been shown to influence the composition of the gut microbiota [39]. Sensitivity analyses revealed that the effect of nut consumption on *Roseburia* was explained by studies involving walnuts. Interestingly, in the study by Tindall and colleagues an additional intervention arm was included, which was not eligible for our meta-analyses. The intervention diet was devoid of walnuts but matched for walnut fatty acids, and this group also experienced an increase in *Roseburia* in comparison to control, suggesting a potential role for lipids in the prebiotic effect of walnuts [27]. In turn, *Roseburia* are suggested to benefit the gastrointestinal environment via their negative association with secondary bile acids following walnut consumption [17]. Secondary bile acids are produced via metabolism of bile salts by bacteria in the gut, and their presence is associated with several disease states [40]. 

The results of meta-analyses of *Roseburia* and *Clostridium* were influenced by nut type. In both cases, when studies involving walnuts were removed from the analyses the results failed to reach significance. Interestingly, *Roseburia* appeared to be affected by dose and duration of intervention, whereby a larger dose of nuts and longer intervention lead to a loss of significance, indicating a potential adaption of the microbiota over time. The study by Dhillon and colleagues [21] explained the results of the meta-analyses of *Dialister, Lachnospira* and *Parabacteroides*. This trial investigated a larger dose, for a longer duration and in a parallel design, in contrast to the other trials in these analyses, as such it is not possible to say which of and to what magnitude these characteristics may contribute to the result. Indeed, sensitivity analyses in this review must be interpreted with caution, due to the small number of studies in the analyses.

When considering the gut microbiota and health, high levels of bacterial diversity are generally associated with positive health outcomes, while a low bacterial diversity is a factor identified in a wide range of disease states [41,42,43,44]. Dietary factors that enhance α-diversity are valuable, in that they might reduce risk and prevent disease in healthy people. Alpha diversity represents the number of unique taxa within a sample. Overall, nut consumption appeared to have no significant effect on any α-diversity metric. However, sensitivity analyses investigating nut type revealed an increase in Shannon index following almond consumption that was just significant. Shannon index is an α-diversity metric that takes into account the number of unique species in sample as well as their relative abundance [28]. As discussed previously, due to their unique food matrix nuts are considered capable of delivering a rich supply of nutrients to the colon for use by the microbiota. This has been most extensively researched in almonds, which have been found to have very small cells [45]. Researchers investigating the bioaccessibility of almonds have identified intact and partially ruptured almond cells in the faecal samples of volunteers on an almond rich diet, and in some cases bacteria were identified infiltrating partially ruptured cells, which appeared to be devoid of intracellular lipid droplets, suggesting the intracellular contents had been utilised by the microbiota [14]. The combination of these factors might explain the observed almond specific effect on α-diversity. 

Beta diversity represents the taxa present between samples; in dietary studies this usually represents the difference in bacteria present before and after an intervention. Due to the nature of reporting of β-diversity measures, it was not possible to conduct meta-analyses. Comparing studies by nut type, the impact of almonds on β-diversity is inconclusive. However, nut consumption had a significant effect on β-diversity in two out of three trials that investigated walnut consumption in healthy adults [16,17]. The trial that reported no effect of walnut consumption on β-diversity was conducting in adults at increased cardiovascular risk [27]. Previous studies have described reduced faecal microbiota diversity in pre-hypertensive adults [46], and obese patients [42], which may explain the findings of this study. 

Gut microbiota composition contributes to optimal gut function and gut symptoms [7]. Despite early indications of a prebiotic effect, few studies have evaluated the impact of nuts on functional or clinical outcomes. Meta-analysis was possible for stool output only, which was not affected by nut consumption. As trials included in the meta-analysis recruited healthy people, this is not a surprising outcome. Future studies may wish to address the impact of nut consumption on gut microbiota and stool output in populations experiencing altered gut microbiota, and sub-optimal gut function such as in constipation [47].

Strengths of this systematic review include the use of the PRISMA [24] and the Cochrane handbook [23] to design a robust search strategy. The protocol was designed and published prior to conducting searches in order to remove reviewer bias. The search strategy was designed to be broad in order to include studies in all participants not experiencing major organ dysfunction. As a consequence, one eligible study was conducted in patients with IBS-D, who are known to experience altered gut microbiota and gut function [48]. This study was not reported in sufficient detail for inclusion in the meta-analyses, however it is one of two studies reporting gut symptoms and has been included in the narrative review. 

Limitations were present at the study and outcome levels. There was poor reporting of the relevant outcomes across studies, in many cases outcomes were measured but could not be included in the meta-analysis due to issues with data format or results described in narrative form only. Methodology between trials varied considerably, including differences in primers used to target the 16S rRNA region of microbial DNA and the use of different databases to assign taxonomy, both of which limit the direct comparability of results between studies. Nuts were provided in various forms, including roasted, chopped, and ground. Mechanical disruption of cells by chopping or grinding to butter is likely to enhance nutrient availability for digestion in the upper gastrointestinal tract, altering the proportion of nutrients that reach the colon intact for use by the microbiota [49,50,51,52,53]. In addition, high temperatures during roasting or cooking are known to influence the polyphenol composition of plant-based foods [54]. Seven of the nine eligible RCTs were crossover design. This design is not ideal for measuring changes in the microbiota following nut consumption, which have been found to persist for up to two weeks following discontinuation of the intervention [15]. Seven of the nine eligible RCTs measured microbiota as a secondary outcome, and so are not powered to detect changes in these outcomes, which therefore highlights the utility of performing meta-analysis here. 

At the review level, the main weakness is the small number of studies that have been included. Overall, little heterogeneity was detected throughout the meta-analyses, however it should be noted that three of the studies included in the meta-analyses were conducted at the same institution, by the same research team and so methods and participant groups are likely to be similar [17,19,20]. Two of these studies contributed four intervention groups each to the meta-analyses [19,20]. 

## 5. Conclusions

Nut consumption affects gut microbiota composition at the genus level, but not phyla or diversity, however nut type and, to some extent, duration of consumption influence the effects. The quality of the included trials was poor, with no studies experiencing low risk of bias across all domains. Overall, the strength of evidence from the meta-analyses is weak. Future parallel design RCTs, powered to detect changes in primary outcomes related to the gut microbiota and incorporating clinical and functional outcomes, are needed, in order to gain robust conclusions on the impact of nuts on the gut microbiota and gut health. 

## Figures and Tables

**Table 1:** Inclusion and exclusion criteria and data extracted for eligible studies using the PICOS ^1^ approach.

PICOS	Inclusion and Exclusion Criteria	Data Extraction
Participants	Adults who were healthy/experiencing minor organ dysfunction not requiring inpatient care were included. Trials comparing clinical populations to healthy populations were excluded unless the impact of nut consumption on the healthy population alone could be extracted. Trials exclusively in children, animals, ex vivo or in vitro were excluded. There were no restrictions for sex or ethnicity.	Age, sex, inclusion and exclusion criteria, number of participants randomised to control and intervention groups.
Interventions	Interventions consisting of a minimum dose of 7 g of tree nuts and/or peanuts per day [25] for a minimum duration of 1 week were eligible. Trials administering nuts with or without skins were eligible as were trials consisting of processed nuts provided the nuts had not been mixed with additional ingredients. Trials administering individual nut components alone, e.g., skins/oils were excluded. Eligible trials were those in which the nuts were provided to participants; interventions consisting of dietary advice to increase nut consumption were excluded. Mixed interventions were excluded unless it was possible to extract the effect of nut consumption alone. Trials with multiple intervention arms were included.	Nut type, dose, frequency, duration of intervention, presentation, processing, instructions for consumption.
Comparators	Trials comparing consumption of nuts to a control involving consumption of no nuts were included. When trials were conducted in controlled feeding environments, only those in which control and intervention diets were matched in energy were included.	Type and dose of comparator, nutrient composition of intervention and control foods.
Outcomes	Trials reporting outcomes relating to faecal microbiota, such as composition or outcomes assessing bacterial activity, were included. Trials reporting clinical subjective or objective measures of gut function including gut transit time, stool form and frequency or gut symptoms were included.	Outcomes measured, method of measurement, baseline and endpoint values or change from baseline. Adverse events and compliance.
Study design	Only randomised controlled trials, utilising parallel or crossover designs, were eligible. If crossover design was used only trials with a washout period were eligible to limit carryover effects. Studies conducted in controlled feeding or free-living environments were eligible.	Study design, duration of washout period, intention to treat analysis, number of excluded participants, reasons for exclusion, randomisation and blinding methods.

^1^ PICOS, participants, intervention, comparator, outcome, study design.

**Table 2:** Characteristics of randomised controlled trials investigating the impact of nut consumption on gut health ^1^.

Study	Participants	Sample Size (%female)	Nut Type	Dose (g/d)	Duration	Comparator	Outcomes Included in Meta-Analysis	Trial Design	Washout
Bamberger 2018 [16]	Healthy adults	142 (64.7)	Walnuts	43	8 wk	Western style control—dietary advice	Microbiota	Crossover Free-living	4 wk
Burns 2016 [18]	Healthy adults	29 (82.8)	Almonds	42.5	3 wk	Usual diet (avoid nuts)	Microbiota, stool frequency	Crossover Free-living	6 wk
Darvishmogh-adam 2019 [22]	IBS-D—Rome IV criteria	50 (58.0)	Almonds	40	20 d	Wheat	None	Parallel Free-living	NA
Dhillon 2019 [21]	Healthy adults	73 (56.2)	Almonds	56.7	8 wk	Graham crackers	Microbiota	Parallel Free-living	NA
Holscher 2018a [17]	Healthy adults	18 (44.4)	Walnuts	42	3 wk	Base diet (food provided)	Microbiota	Crossover Controlled feeding	1 wk
Holscher 2018b [19]	Healthy adults	18 (44.4)	Almonds (whole natural, whole roasted, chopped, butter)	42	3 wk	Base diet (food provided)	Microbiota	Crossover Controlled feeding	1 wk
Tindall 2020 [27]	Adults at risk of cardiovascular disease	42 (45.2)	Walnuts	57–99 g (18% daily energy intake)	6 wk	Base diet with ALA from walnut matched for oleic acid	Microbiota	Crossover Controlled feeding	Mean: 23 d Range: 1–164 d
Ukhanova 2014 [20]	Healthy adults	Almond 18 (44.4) Pistachio 16 (50.0)	Almond OR Pistachio	42 OR 84	18 d	Base diet (food provided)	Stool frequency	Crossover Controlled feeding	Almond: 1 wk Pistachio: 2 wk

^1^ IBS-D, diarrhoea predominant irritable bowel syndrome; d, day; wk, week; g/d, gram per day.

**Table 3:** Results of meta-analyses comparing nut consumption with control on relative abundance of bacterial taxa at the phylum and genus levels, alpha diversity metrics and stool frequency ^1^.

Outcome	No. of Studies in the Meta-Analysis (Ref)	Results			Heterogeneity		
		Participants *n*	Meta-Analysis Overall Estimate (95% CI)	*P*	Chi-Square Test	*P*	*I*^2^ (%)
**Phyla**							
p_Actinobacteria	4 [17,18,19,21]	138	−0.14 (−0.42, 0.14)	0.32	6.45	0.37	7
p_Bacteroidetes	4 [17,18,19,21]	138	0.19 (−0.07, 0.45)	0.16	1.15	0.98	0
p_Firmicutes	4 [17,18,19,21]	138	0.04 (−0.22, 0.30)	0.76	3.25	0.78	0
p_Proteobacteria	4 [17,18,19,21]	138	−0.07 (−0.51, 0.38)	0.77	14.3	**0.03**	58
p_Tenericutes	2 [18,21]	102	−0.00 (−0.35, 0.34)	0.98	0.74	0.39	0
p_Verrucomicrobia	4 [17,18,19,21]	138	−0.20 (−0.46, 0.06)	0.13	0.78	0.99	0
**Genus**							
g_Faecalibacterium	4 [17,19,21,27]	151	0.11 (−0.16, 0.38)	0.43	6.45	0.37	7
g_Roseburia	4 [17,19,21,27]	151	0.36 (0.10, 0.62)	**0.006**	4.81	0.57	0
g_Streptococcus	4 [17,19,21,27]	151	−0.02 (−0.27, 0.24)	0.91	0.08	1	0
g_Blautia	4 [16,17,19,21]	251	−0.15 (−0.34, 0.03)	0.11	2.25	0.89	0
g_Bifidobacteria	4 [16,17,19,21]	251	−0.09 (−0.39, 0.21)	0.56	9.31	0.16	36
g_Coprococcus	3 [17,19,21]	109	−0.10 (−0.41, 0.20)	0.52	0.77	0.98	0
g_Lachnospira	3 [17,19,21]	109	0.33 (0.02, 0.64)	**0.03**	1.11	0.95	0
g_Ruminococcus	3 [17,19,21]	109	−0.10 (−0.40, 0.21)	0.54	1.38	0.93	0
g_Dorea	3 [17,19,21]	109	−0.08 (−0.39, 0.22)	0.59	1.24	0.94	0
g_Clostridium	3 [17,19,21]	109	0.40 (0.10, 0.71)	**0.01**	0.86	0.97	0
g_Oscillospira	3 [17,19,21]	109	−0.10 (−0.42, 0.22)	0.55	5.25	0.39	5
g_Dialister	3 [17,19,21]	109	0.44 (0.13, 0.75)	**0.005**	1.33	0.93	0
g_Bacteroides	3 [17,19,21]	109	0.08 (−0.23, 0.38)	0.61	0.23	1	0
g_Parabacteroides	3 [17,19,21]	109	−0.31 (−0.62, −0.00)	**0.05**	2.16	0.83	0
g_Collinsella	3 [17,19,21]	109	−0.16 (−0.46, 0.15)	0.32	0.92	0.97	0
g_Akkermansia	3 [17,19,21]	109	−0.21 (−0.51, 0.10)	0.18	0.77	0.98	0
g_Anaerostipes	3 [16,17,21]	233	0.09 (−0.47, 0.64)	0.75	9.79	**0.007**	80
g_Phascolarctobacterium	2 [19,21]	91	0.16 (−0.19, 0.50)	0.37	0.75	0.94	0
g_Prevotella	2 [19,21]	91	0.14 (−0.21, 0.48)	0.44	0.44	0.98	0
**Alpha diversity**							
Chao-1 index	3 [17,19,21]	109	0.23 (−0.07, 0.54)	0.14	0.83	0.98	0
Observed OTUs	3 [17,19,21]	109	0.26 (−0.05, 0.56)	0.10	0.40	1	0
Shannon index	3 [16,18,21]	244	0.14 (−0.23, 0.51)	0.45	5.27	0.07	62
Simpson index	2 [16,21]	215	−0.16 (−0.37, 0.05)	0.13	0.60	0.44	0
Whole tree	2 [17,19]	36	−0.01 (−0.42, 0.40)	0.96	0.55	0.97	0
**Stool frequency ^2^**	3 [18,20]	63	0.04 (−0.11, 0.18)	0.61	1.23	0.87	0

Data were meta-analysed using a random-effects model and are presented as standardised mean difference unless otherwise specified. *P* values in bold are statistically significant (*P* < 0.05). ^1^ OTU, operational taxonomic unit; ^2^ Meta-analysis overall estimate is weighted mean difference (95% CI).

## Supplementary Materials

The following are available online at https://www.mdpi.com/2072-6643/12/8/2347/s1, Note S1: Example search strategy for MEDLINE (Ovid), Table S1: Sensitivity analyses.
