# Supplementary material for: Nuts and their Effect on Gut Microbiota, Gut Function and Symptoms in Adults: A Systematic Review and Meta-Analysis of Randomised Controlled Trials
Source: Nutrients. 2020 Aug 6;12(8):2347. doi: 10.3390/nu12082347 (PMC7468923; doi:10.3390/nu12082347)
Supplement: Supplementary file 1 [file nutrients-12-02347-s001.pdf]

Supplementary note 1: Example search strategy for MEDLINE (Ovid)

1. Nut.mp
2. Nuts.mp
3. Almond.mp
4. Prunus dulcis.mp
5. Brazil nut.mp
6. Bertholletia.mp
7. Cashew.mp
8. Anacardium.mp
9. Chestnut.mp
10. Castanea.mp
11. Hazelnut.mp
12. Corylus.mp
13. Macadamia.mp
14. Marking nut.mp
15. Semecarpus anacardium.mp
16. Pecan.mp
17. Carya.mp
18. Pine nut.mp
19. Pinus.mp
20. Pistachio.mp
21. Pistachia.mp
22. Sachi inchi nut.mp
23. Plukenetia volubilis.mp
24. Walnut.mp
25. Juglans.mp
26. Ground nut.mp
27. Bambara groundnut.mp
28. Vigna subterranean.mp
29. Kersting's nut.mp
30. Macrotyloma geocarpum.mp
31. Peanut.mp
32. Arachis.mp
33. exp Nuts/
34. exp Prunus/ or exp Prunus dulcis/
35. exp Bertholletia/
36. exp Anacardium/
37. exp Corylus/
38. exp Macadamia/
39. exp Semecarpus/
40. exp Carya/
41. exp Pinus/
42. exp Pistachia/
43. exp Juglans/
44. exp Arachis/
45. 1 or 2 or 3 or 4 or 5 or 6 or 7 or 8 or 9 or 10 or 11 or 12 or 13 or 14 or 15 or 16 or 17 or 18 or 19 or 20 or 21 or 22 or 23 or 24 or 25 or 26 or 27 or 28 or 29 or 30 or 31 or 32 or 33 or 34 or 35 or 36 or 37 or 38 or 39 or 40 or 41 or 42 or 43 or 44
46. Gut microbiome.mp
47. Gut microbiota.mp

48. Gut bacteria.mp
49. Gut flora.mp
50. Short-chain fatty acids.mp
51. SCFA.mp
52. Acetate.mp
53. Proprionate.mp
54. Butyrate.mp
55. Stool pH.mp
56. Gut transit.mp
57. Gut transit time.mp
58. Transit time.mp
59. Stool frequency.mp
60. Stool consistency.mp
61. Stool weight.mp
62. Gut symptoms.mp
63. exp Gastrointestinal microbiome/
64. exp Microbiota/
65. exp Fatty acids, Volatile/
66. exp Acetates/
67. exp Butyrates/
68. exp Feces/
69. exp Gastrointestinal Transit/
70. exp Gastrointestinal Motility/
71. exp Constipation/
72. exp Diarrhea/
73. exp Irritable Bowel Syndrome/
74. 46 or 47 or 48 or 49 or 50 or 51 or 52 or 53 or 54 or 55 or 56 or 57 or 58 or 59 or 60 or 61 or 62 or 63 or 64 or 65 or 66 or 67 or 68 or 69 or 70 or 71 or 72 or 73
75. 45 AND 74
76. exp animals/ not humans.sh
77. 75 NOT 76

Supplementary Table 1: Sensitivity analyses

Sensitivity analyses for outcomes where significant heterogeneity was observed, or where the meta-analysis was significant.

Table S1. Results of sensitivity analyses for characteristics influencing the effect of nut consumption on gut microbiota composition and diversity in adults[1].

Subgroup	No. of studies (ref)	Results: Participants *n*	Results: Meta-analysis overall estimate (95% CI)	Results: *P*	Heterogeneity: Chi-square test	Heterogeneity: *P*	Heterogeneity: I^2 (%)
**p_Proteobacteria**							
Almond	3 [18,19,21]	120	-0.20 (-0.66, 0.25)	0.38	9.91	0.08	50
Duration < 4 weeks<br>Dose < 45 g/d<br>Crossover design	3 [17–19]	65	-0.20 (-0.67, 0.28)	0.42	9.41	0.09	47
**g_Clostridium**							
Almond	2 [19,21]	91	0.34 (-0.01, 0.68)	0.06	0.18	1	0
Duration < 4 weeks<br>Dose < 45 g/d<br>Crossover design	2 [17,19]	36	0.47 (0.06, 0.88)	**0.03**	0.63	0.96	0
**g_Dialister**							
Almond	2 [19,21]	91	0.44 (0.09, 0.79)	**0.01**	1.33	0.86	0
Duration < 4 weeks<br>Dose < 45 g/d<br>Crossover design	2 [17,19]	36	0.33 (-0.08, 0.74)	0.12	0.66	0.96	0
**g_Lachnospira**							
Almond	2 [19,21]	91	0.39 (0.04, 0.74)	**0.03**	0.61	0.96	0
Duration < 4 weeks<br>Dose < 45 g/d<br>Crossover design	2 [17,19]	36	0.30 (-0.11, 0.71)	0.15	1.05	0.90	0
**g_Parabacteroides**							
Almond	2 [19,21]	91	-0.43 (-0.77, -0.08)	**0.02**	0.19	1	0

Duration < 4 weeks<br>Dose < 45 g/d<br>Crossover design	2 [17,19]	36	-0.20 (-0.61, 0.21)	0.35	1.48	0.83	0
**g_Roseburia**							
Non-clinical participants	3 [17,19,21]	109	0.24 (-0.06, 0.55)	0.12	2.84	0.72	0
Almond	2 [19,21]	91	0.19 (-0.16, 0.53)	0.29	2.39	0.66	0
Walnut	2 [17,27]	60	0.58 (0.19, 0.97)	**0.004**	0.26	0.61	0
Duration < 4 weeks<br>Dose < 45 g/d	2 [17,19]	36	0.43 (0.02, 0.84)	**0.04**	1.05	0.9	0
Duration > 4 weeks<br>Dose > 45 g/d	2 [21,27]	115	0.33 (-0.31, 0.96)	0.31	3.58	0.06	72
Crossover design	3[17,19,27]	78	0.52 (0.21, 0.84)	**0.001**	1.53	0.91	0
**g_Bifidobacteria**							
Almond	2 [19,21]	91	-0.28 (-0.62, 0.07)	0.11	0.28	0.99	0
Walnut	2 [16,17]	160	0.07 (-0.53, 0.66)	0.83	3.10	0.08	68
Duration < 4 weeks	2 [17,19]	36	-0.35 (-0.76, 0.06)	0.09	0.10	1	0
Duration > 4 weeks	2 [16,21]	215	0.09 (-0.41, 0.58)	0.74	3.77	0.05	74
Dose < 45 g/d<br>Crossover design	3 [16,17,19]	178	-0.07 (-0.43, 0.28)	0.69	7.44	0.19	33
**g_Anaerostipes**							
Walnut<br>Dose < 45 g/d<br>Crossover design	2 [16,17]	160	-0.15 (-0.61, 0.30)	0.51	2	0.16	50
Duration > 4 weeks	2 [16,21]	215	0.06 (-0.71, 0.83)	0.88	8.84	0.003	89
**Alpha Diversity – Shannons index**							
Almond	2 [18,21]	102	0.35 (0.00, 0.70)	0.05	0.70	0.40	0
Duration > 4 weeks	2 [16,21]	215	0.15 (-0.42, 0.72)	0.60	4.89	0.03	80
Dose < 45 g/d<br>Crossover design	2 [16,18]	171	-0.05 (-0.27, 0.17)	0.63	1.02	0.31	2

[1]Data were meta-analysed using a random-effects model and are presented as standardised mean difference. g/d, grams per day.
